# Patient engagement in health implementation research: A logic model

**DOI:** 10.1111/hex.13782

**Published:** 2023-06-12

**Authors:** Mathieu Bisson, Kris Aubrey‐Bassler, Maud‐Christine Chouinard, Shelley Doucet, Vivian R. Ramsden, Olivier Dumont‐Samson, Dana Howse, Mireille Lambert, Charlotte Schwarz, Alison Luke, Norma Rabbitskin, André Gaudreau, Jude Porter, Donna Rubenstein, Jennifer Taylor, Mike Warren, Catherine Hudon

**Affiliations:** ^1^ Département de médecine de famille et de médecine d'urgence Université de Sherbrooke Sherbrooke Québec Canada; ^2^ Primary Healthcare Research Unit, Faculty of Medicine Memorial University St. John's Newfoundland and Labrador Canada; ^3^ Faculty of Nursing Université de Montréal Montreal Québec Canada; ^4^ Department of Nursing and Health Sciences University of New Brunswick Fredericton New Brunswick Canada; ^5^ Department of Academic Family Medicine University of Saskatchewan Saskatoon Saskatchewan Canada; ^6^ Patient Partners QC, NS, NB, NL Canada

**Keywords:** health implementation research, logic model, patient engagement, patient involvement, patient partnership in research

## Abstract

**Introduction:**

Growing evidence supports patient engagement (PE) in health implementation research to improve the quality, relevance and uptake of research. However, more guidance is needed to plan and operationalize PE before and throughout the research process. The aim of the study was to develop a logic model illustrating the causal links between context, resources, activities, outcomes and impact of PE in an implementation research programme.

**Methods:**

The Patient Engagement in Health Implementation Research Logic Model (hereafter the Logic Model) was developed using a descriptive qualitative design with a participatory approach, in the context of the PriCARE programme. This programme aims to implement and evaluate case management for individuals who frequently use healthcare services in primary care clinics across five Canadian provinces. Participant observation of team meetings was performed by all team members involved in the programme and in‐depth interviews were conducted by two external research assistants with team members (*n* = 22). A deductive thematic analysis using components of logic models as coding categories was conducted. Data were pooled in the first version of the Logic Model, which was refined in research team meetings with patient partners. The final version was validated by all team members.

**Results:**

The Logic Model highlights the importance of integrating PE into the project before its commencement, with appropriate support in terms of funding and time allocation. The governance structure and leadership of both principal investigators and patient partners have significant effects on PE activities and outcomes. As an empirical and standardized illustration that facilitates a shared understanding, the Logic Model provides guidance for maximizing the impact of patient partnership in various contexts for research, patients, providers and health care.

**Conclusion:**

The Logic Model will help academic researchers, decision makers and patient partners plan, operationalize, and assess PE in implementation research for optimal outcomes.

**Patient or Public Contribution:**

Patient partners from the PriCARE research programme contributed to developing the research objectives and designing, developing and validating data collection tools, producing data, developing and validating the Logic Model and reviewing the manuscript.

## 1 INTRODUCTION

Literature on patient engagement (PE) in research has increased exponentially in the last decade. The many benefits of having patients as partners in research (hereafter patient partners) are well documented. PE can improve the quality of research^1^, ^2^, ^3^, ^4^ by co‐designing the study's protocols, choosing relevant outcomes,^5^ improving processes and ethical practises,^2^, ^4^ as well as validating research instruments.^4^ PE can also increase study enrolment.^5^ Academic researchers who involve patients in research recognize patients' experience as expertise.^4^ Based on patients' priority and holistic needs assessment,^6^ this strategy can improve the relevance and uptake of research.^1^, ^6^, ^7^ PE is more effective when patients with lived experience are meaningfully involved as research team members.^8^ Involving patients in key aspects of implementation research can also facilitate and enhance implementation processes,^9^ which can improve outcomes for both the research process and patient healthcare.^2^ Patients' perspectives can produce innovative solutions that improve the health and well‐being of the population.^7^, ^10^ PE has positive impacts on researchers and patient partners such as enhanced skills, and increased self‐confidence, social support, learnings and satisfaction.^2^, ^4^, ^7^

Many tools and frameworks have been proposed to assess PE in implementation research.^11^, ^12^, ^13^ In a systematic review that includes 65 frameworks, Greenhalgh et al.^14^ classified them into five categories: power‐focused; priority‐setting; study‐focused; report‐focused and partnership‐focused. In another systematic review that included 14 models and frameworks, Chudyk et al.^15^ organised elements underlying PE in health service research into six categories: principles; foundational components; context; actions; levels and outcomes. For academic researchers and patient partners, these PE frameworks are useful to identify the essential components of their programme, but do not necessarily provide the ‘recipe’ linking, in operational terms, the principles, strategies, outcomes and impacts.^16^ Logic models aim to provide a systematic way to visualize the interaction between the rationale of an intervention, planned activities, required resources and expected outcomes,^17^ and offer an interesting means to advance our knowledge about this ‘recipe’. Logic models can support the reporting and standardization of PE in research^18^ by explaining the ‘how’ and ‘what’ of PE in implementation research.^19^ For example, Merker et al.^20^ proposed a logic model to articulate the activities being implemented to support PE and its anticipated outcomes in the specific context of veteran engagement. Developing a logic model about PE in a broader context of implementation research could be useful.

The objective of the study was to develop a logic model illustrating empirically the causal links between context, resources, activities and expected outcomes of PE in health implementation research.

## 2 METHODS

### 2.1 Settings: PriCARE research programme

This study focused on the engagement of patient partners deployed in the PriCARE research programme, which is detailed elsewhere.^21^, ^22^ PriCARE implemented and evaluated a case management intervention for individuals that frequently use healthcare services in primary care clinics across five Canadian provinces: New Brunswick, Newfoundland, Labrador, Nova Scotia, Quebec, and Saskatchewan.

One to two patient partners were recruited in each participating province to work closely with the provincial research team. Each province circulated a posting to different networks where interested patient partners could apply and then meet with the local principal investigator and coordinator. In addition to taking an active part in the various steps of the research process, from the proposal stage to knowledge transfer activities, the patient partners participated in both the central decision‐making committee as well as a ‘community of practice’ to foster their engagement in various stages of the research programme and ensure that their priorities were considered.

### 2.2 Design

A descriptive qualitative design^23^ was conducted with a participatory approach^24^ involving patient partners and academic researchers of the PriCARE programme. As some of the academic researchers were also healthcare providers, the perspective of this category of participant was included.

### 2.3 Sampling and participants

All the PriCARE team members were invited to participate in this study using purposeful sampling.^25^ Twenty‐two members agreed to participate including principal investigators (*n* = 5); co‐investigators (*n* = 2); research coordinators and assistants (*n* = 8); one postdoctoral researcher; patient partners (*n* = 7) from four out of the five participating Canadian provinces. All participants discussed the aim of the current study during Steering Committee meetings and patient partners' Community of Practice meetings.

### 2.4 Data collection

Participant observation of team meetings was performed by the team members involved in the PriCARE research programme, from November 2018 to February 2021. The meetings observed were a monthly half‐hour Community of Practice meeting including six patient partners and five research coordinators and assistants, and a monthly 1‐h Steering Committee meeting including the same patient partners and research coordinators and assistants, as well as eight co‐investigators and one postdoctoral researcher.

Individual semistructured interviews were conducted with team members who agreed to participate and who attended regular team meetings. Two research assistants external to the PriCARE research programme conducted the interviews to avoid social desirability bias and self‐censorship. The interview guide was adapted to each category of participants (patients and research staff), and patient partners contributed to its design. Based on the categories of a classic logic model, questions were about resources allocated to support PE, types of research activities that participants were involved in or should have taken place to improve patients' contribution to the research, perceptions of the value that PE added to the research programme and expected outcomes of PE in the research programme. Other topics discussed included the role of team members who participated in the research programme; mechanisms put in place to support PE; opportunities for interaction and feedback amongst team members to support PE; team members' expectations of PE when they joined the research programme; potential activities, events or incidents related to PE and the contribution that team members would like to make in the future. Sociodemographic data (gender, age, location, first language, time of involvement in the PriCARE programme) were also collected so that the participants could be described. Since the ‘context’ component of the logic model was well described in the Canadian Institutes of Health Research SPOR UNITS document,^3^ it was not explored during the interviews. (‘SPOR’ is the Canadian Institutes for Health Research Strategy for Patient‐Oriented Research, which has formed funding partnerships with provinces and territories, philanthropic organisations, academic institutions and health charities. SPOR funds 10 SUPPORT Units across Canada to provide specialized services to researchers, patients, clinicians, policy makers and SPOR‐funded entities to conduct patient‐oriented research). Interviews with academic team members were digitally recorded and transcribed verbatim. To preserve the confidentiality of the patient partners, the two external assistants produced a deidentified summary of their interviews that was validated at a meeting in which the patient partners reviewed and approved the summary.

### 2.5 Analysis

Data were analyzed using a deductive thematic analysis approach^26^ where the themes corresponded to the categories of a classic logic model (i.e., resources, activities, outputs, outcomes, impact). All data were categorized under these themes using NVivo 12 software by research coordinators and assistants involved in the PriCARE programme with expertise in qualitative research. Data about resources, activities, output, outcomes and impact were pooled^26^, ^27^ and included in a first version of the Logic Model. Team meetings with a principal investigator, a co‐principal investigator, a coordinator, a research assistant and a patient partner helped to refine the Logic Model. It was then presented to all participants during Steering Committee meetings and patient partners' Community of Practice meetings where comments were incorporated. A new version of the Logic Model was then shared with everyone by email for review and final validation following an iterative and participative process.^28^

## 3 RESULTS

Table 1 presents sociodemographic characteristics of the participants. A total of 22 participants (72.7% female) completed individual or group interviews lasting 30–60 min. Seven patient partners and 15 academic research team members participated. Most patient partners were between 55 and 64 years old (57%), while most academic researchers were between 35 and 44 (47%). Most participants spoke English, had training in patient involvement in research, and had previous experience with patient involvement in research. Six academic researchers and two patient partners were involved as early as the grant submission stage.

**Table 1:** Sociodemographic characteristics of the participants (*N* = 22).

	Academic researchers (*n* = 15)	Patient partners (*n* = 7)
	*n* (%)	*n* (%)
Gender		
Female	11 (73.3)	5 (71.4)
Age (years)		
25–34	2 (13.3)	0 (0.0)
35–44	7 (46.7)	0 (0.0)
45–54	4 (26.7)	2 (28.6)
55–64	0 (0.0)	4 (57.1)
≥65	2 (13.3)	1 (14.3)
Location		
Newfoundland and Labrador	2 (13.3)	1 (14.3)
New Brunswick	4 (26.7)	2 (28.6)
Nova Scotia	3 (20.0)	2 (28.6)
Quebec	6 (40.0)	2 (28.6)
First language		
English	10 (66.7)	5 (71.4)
French	5 (33.3)	2 (28.6)
Time of involvement in PriCARE		
Since grant submission	6 (40.0)	2 (28.6)
Since initial implementation	5 (33.3)	0 (0)
Joined during the implementation	3 (20.0)	5 (71.4)
Joined recently (6 months)	1 (6.7)	0 (0.0)
Had PE in research training	8 (53.3)	4 (57.1)
Had previous PE in research experience	10 (66.7)	5 (71.4)

	Mean (SD)	Mean (SD)
Years of PE experience in research^a^	5.8 (2.6)	10.5 (8.4)

Abbreviations: PE, patient engagement; SD, standard deviation.

^a^ Only for participants who had previous patient engagement experience in research (academic researcher *n* = 5; patient partner *n* = 4).

**Figure 1:**
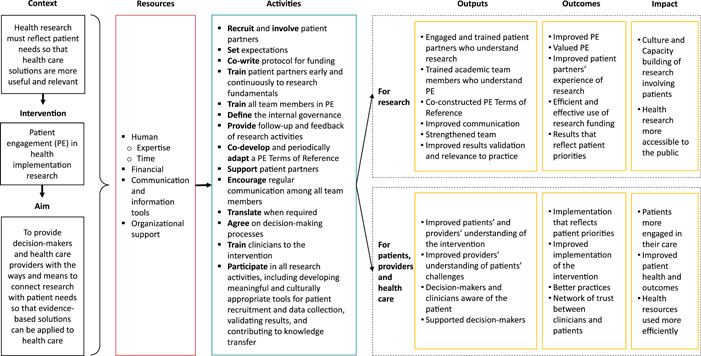
Patient engagement in health implementation research logic model.

Figure 1 presents the linkages between context, resources, activities, outputs, outcomes and the impact of PE in research.

### 3.1 Resources

Both material and nonmaterial resources were mobilized in the PriCARE programme to support PE. ‘Human resources’ include all partners who contributed to supporting PE: principal investigators (some being healthcare providers); research coordinators and assistants and patient partners. Research assistants and coordinators were especially involved.

Patient partners identified human resources as resources allocated to ensure patient partners' engagement in the program. All research officers (research assistants, coordinators) are available to them to ensure that they do not feel like separate members, but rather full members of the research team. (Summary of patient partner interviews)

Team members had a range of experience with patient‐oriented research within other research teams and projects, which fostered a common understanding of the goals and value of PE as well as how to work efficiently as a team. Patient partners brought their personal experiences to healthcare, which enriched academic team members' understanding of the issues. Moreover, some patient partners had considerable experience in research as well as skills to facilitate and coordinate activities (e.g., community of practice planning).

Patient partners who have experience analyzing data in other projects can really bring in ideas that we didn't have, angles that we didn't have, things that we didn't see, things that we might have tamped down but ultimately decided to keep. (Investigator #2)

Some patient partners had previous research experience and felt more comfortable with their knowledge of the research process. (Summary of patient partner interviews)

Lead members of the research team (e.g., a co‐principal investigator) demonstrated support for PE, modelling what PE means in practice. Through the course of the research programme, which is in its third year in the spring of 2023, there have been many learnings and improvements that brought the experience and skills of the team members closer together.

A few patient partners mentioned that in several areas, significant progress has been made in having regular dialogue between patient partners and researchers. This included meaningful exchange of ideas and collaborative problem solving. […] Patient partners expressed that this was a learning experience for everyone on the research team. Though at first, they did not feel their voice was being heard or appreciated by all, they eventually worked through a process and co‐designed a solution. (Summary of patient partner interviews)

PE support required a significant amount of time, especially for the lead coordinator and local coordinators who were in close contact with patient partners.

Like how do I manage the time so that it's optimal for everybody? So as a coordinator, that's the sort of stuff that I'm thinking through. (Staff #5)

It takes extra time to explain our thoughts, our rationale, our reasoning, and, you know, it makes us think twice about the decisions we're making. (Investigator #11)

A majority of patient partners stated that a lot of time is spent in meetings and/or reviewing emails with different Program documents. (Summary of patient partner interviews)

This time was spent completing various activities, as well as allowing trust and relationships to grow.

When we don't know each other, well, that takes time. There are patients who are partners, it's been a long time, and we don't know each other because we only see each other in the Communities of Practice, and we don't talk about our personal lives. (Staff #1)

In addition to ‘human resources’, ‘financial resources’ were allocated to recognize patient partners' valuable expertise and contributions, and the required funding had been planned in the initial budget of the grant proposal.

The PE was planned from the beginning, and it was budgeted. We believe in it, we think it is important, so we find ways to adjust and to be able to recognize PE in terms of time invested. (Investigator #6)

‘Communication resources’ in the Logic Model refer to technologies and tools used to share information and to ensure communication between patient partners and academic team members (email, telephone, online software or web applications to meet virtually).

Sometimes we have things to talk about, so instead of sending emails, we say to each other ‘Can we talk for 10 minutes online?’ So, we've used it a lot for our communication. (Staff #1)

‘Organizational resources’ refer to provincial SPOR SUPPORT units^3^ (Newfoundland and Labrador, Maritime and Quebec) that helped with the training and recruitment of patient partners. The universities in each province also provided facilities and financial services (help in budget management, including patient partner payments), as well as information and technology services that supported the use and installation of software and communication tools.

### 3.2 Activities

Both patient partners and academic researchers were involved in all research activities from project inception to knowledge translation. Since PE was integrated into the governance structure of the research programme, ongoing activities related to PE support such as involving patient partners, defining roles, setting expectations, communicating between team members and participation in decision‐making were not different from typical research activities.

I would say there's been a very highly participative group, as you know, functioning independently as well as together with the rest of us as well to really offer advice and support. And even independently, you know, not just providing feedback on stuff the researchers initiate, but also, I would say, initiating some ideas of their own and input of their own. (Investigator #15)

A co‐development approach facilitated patient partners providing their feedback and support at any time, by email or telephone, during virtual team meetings, Community of Practice or ad hoc committee meetings. A PE Terms of Reference document aiming to describe patient partners' roles within the governance structure was co‐developed and periodically adapted in collaboration with patient partners and academic researchers.

The PE Terms of Reference described how patient engagement worked within the team. So, whenever we recruited a new team member, we shared it with them. It was helpful because it gave a more global view of patient engagement in the project. (Investigator #2)

Patient partners' Community of Practice facilitated discussion of topics of particular interest or importance to them, or for which they have been mandated by the Steering Committee, and to later share some or all aspects of those discussions with the larger team. Patient partners' involvement in activities varied according to their availability and interests.

Patient partners have participated in different research activities based on their time involved in the work and the ways they would like to be involved in the work. Different patient partners want and expect different levels of engagement based on their interests, skills, availability, and lived experience. (Summary of patient partner interviews)

Some patient partners took on a leadership role such as the facilitation of meetings, communication between patient partners and members of the academic research team and contribution to clinicians' training to help them understand the experience of patients. As the research team was composed of both French and English speakers, translation of documents and discussions (oral or written) was an activity done by the academic researchers when necessary to achieve common understanding and to foster engagement of all research team members, particularly French‐speaking patient partners.

### 3.3 Outputs, outcomes and impact

Participants reported outputs, outcomes and impacts of PE for both the research and for patients, providers and healthcare. Outputs for the research refer to significant patient partners' involvement; common understanding of both the research components and roles and benefits of PE in research; co‐construction of PE Terms of Reference; communication amongst team members; a stronger team and improved validation of results and relevance to practice. For patients and providers, PE in research produces a better understanding of case management as an intervention, for example, an understanding of challenges faced by patients.

The voice of the patients, when you train professionals, has almost more weight than your own voice as a researcher, in the sense that I am the trainer, well, I know what case management is, I am a nurse, but when [name of a patient partner] spoke, it had even more weight, because she expressed it as the patients' experience. (Investigator #2)

PE enhances decision makers' and clinicians' awareness and understanding of the patient perspective and supports decision makers' role by giving relevant direction for the implementation of interventions.

Among the ways patient partners feel their group brings value or will bring value to the Program, there are […] support with decision makers […]. (Summary of patient partner interviews)

The outcomes mostly concern people who were directly involved in the research programme, including case management implementation, that is, research team members, decision makers, clinic managers, clinicians and patients. Regarding research outcomes, the PE activities enhanced the perceived value of PE, improved patient partners' experience of research, supported efficient and effective use of research funding and produced results that reflect patient priorities.

Helping researchers and implementers better understand patient‐related considerations. For example, better understand what matters to patients, developing and delivering the questionnaire, patient recruitment approaches and communication material, bringing organizational skills to the Program, the Terms of Reference, committee organization, and local issues. (Summary of patient partner interviews)

Regarding outcomes for patients, providers and healthcare, PE activities contributed to an implemented intervention that has the potential to reflect patient priorities, an improved implementation of the intervention, better clinical practises (i.e., adapted to the needs of the patients) and a relationship of trust amongst researchers, clinicians, and their patients.

The involvement of patient partners has led us to discover and understand the importance of having the patient's voice in all our work of developing an intervention because, in the end, they are the ones who will receive the intervention. So, they tell us what they need. (Staff #3)

The project can provide better tools for clinicians and physicians, and good collaboration between clinicians and patients would also make it possible to create a network of trust. (Summary of patient partner interviews)

For the research, impacts included building culture and capacity for research involving patients and making health research more accessible to the public.

I think all the members of the research team will go away with a much fuller realization of roles that patient partners can play in these kinds of projects. And that's a huge contribution. That's a capacity building contribution well beyond the study itself, right. (Investigator #10)

We need to build a bridge with the population, create links and then put forward the experience of patients. I see this as a way to make research more accessible to everyone, more democratic. […] In the long run, I think it contributes to the development of a new culture in research. (Staff #4)

Overall, I think the Program has made all team members (PPs, researchers, coordinators) and hopefully Case Managers and clinical staff better at patient‐oriented research and patient engagement. It has been a new approach to health research for most of us. We can all carry our experience to the remainder of the Program and on to our next research projects. (Summary of patient partner interviews ‐ comments directly from individual patient partners)

Concerning impacts related to patients, providers and health care, PE in research facilitates patients being more engaged in their care, may improve patient health and related outcomes, and makes the use of health resources more efficient.

Ultimately, if the objectives of the research program are achieved, the engagement of patient partners will lead to better outcomes for patients with chronic conditions. It should ultimately improve their quality of life, highlight the importance of being involved in their care and lead to more efficient use of healthcare resources, i.e., people, infrastructures, and money. (Summary of patient partner interviews)

## 4 DISCUSSION

The Logic Model presented here illustrates the connexions between resources, activities, and outcomes of PE in health implementation research. As a roadmap, it provides guidance on which resources and activities are required to efficiently plan and operationalize PE in research. As a standardized evaluation tool, the Logic Model can inform which outcomes of PE the research team should focus on (or not), both for the research process and the implementation process of other complex interventions in various settings. Considering the broad context of primary care, the Logic Model may be transferable to other health implementation research contexts in industrialized countries.

Danish et al.'s^29^ study on the description of the PriCARE ‘program logic perspective’ identified resources, processes and relationships (rather than context, resources, activities and outcomes). Their study supported the importance of a governance structure that integrates patient partnerships early in the programme to facilitate the evaluation and continuous improvement of PE. The authors argue that providing a framework for documenting, categorizing, monitoring and improving PE activities throughout the various phases of a research project strengthens PE evaluation capacity.^29^ Boivin et al.^8^ ^,p.2^ concur in an editorial on the importance of rigorous evaluation of the patient and public involvement in research, stating that there is a need to ensure that PE ‘becomes an integral, robustly conducted, and well‐resourced component of research, not a last minute add on’.

Logic models are often criticized for their inability to describe intangible factors such as relationships, collaboration and communication within a research team.^30^ They also have limited capacity to evaluate a programme in a more comprehensive way.^11^ However, Beland et al.'s^31^ study, complementary to ours, mitigates these weaknesses by documenting relationships within the PriCARE team regarding PE from the perspective of both patient partners and academic researchers. ‘Evolving relationships’ described as ‘how partnerships grew and improved over time with an acceptance of tensions and willingness to move beyond them’^31^ can be considered as an outcome of the patient partners' support and regular communication amongst all team members, two activities identified in the Logic Model.

The Logic Model identifies expertise as a necessary human resource for significant PE. According to Danish et al.,^29^ investigators have a major leadership role in supporting the integration of PE activities by modelling positive attitudes and behaviours towards PE, ensuring the availability and the expertise of dedicated personnel to facilitate the management of PE resources, processes and relationships, and ensuring a timely response to challenges. On their side, Beland et al.^31^ identified that patient partners may also play an important leadership role by providing skills to facilitate meetings amongst patient partners.

The Logic Model corroborates other work, highlighting that PE increases the relevance of research by aligning the results and implementing interventions with patient needs and priorities.^3^, ^6^, ^31^, ^32^, ^33^ PE connects research with patient needs so that evidence‐based solutions can be applied in health care.^6^ As mentioned by Duffet,^34^ patient partners may increase transparency and trust in research, which may lead to research that has a greater impact on the ultimate care of patients. These remarks are consistent with the Logic Model, which identifies ‘health research more accessible to the public' and ‘improved patient and health system outcomes’ as PE impacts.

Activities related to PE support take a significant amount of time, which remains an important element to consider in the planning of research projects that involve patients. Interestingly, a mixed methods study by Blackburn et al.^35^ aiming to explore the extent, quality and impact of patient and public involvement in research, and a systematic review by Domecq et al.^5^ on how to best conduct PE in healthcare research, and reported that the main challenge of PE for researchers is time. Funding needed for appropriate compensation for PE in research was also reported.^5^ The Logic Model highlights time and funding as necessary resources for meaningful PE. Further research should be developed to better understand the dynamics between the time required to support PE, the funding required for this work and research team members' expertise, in relation to PE outcomes.

The data collection methods used in the current study (participant observation, in‐depth interviews and dyadic approach) have also helped fill the gap concerning the need for more systematic data collection^30^ and the need to assess PE from the perspective of both patient partners and academic researchers.^11^

### 4.1 Limitations

A limitation of this study is that patient partners and academic research team members acting as study participants and contributing to their own data analysis could potentially cause bias due to social desirability and the risk of self‐censorship. However, external research assistants hired to collect data mitigate this limitation. The participatory approach and the active role of participants in data analysis and interpretation provide some strengths because of their familiarity with the research programme. Furthermore, the team members are also involved in many other projects involving PE and bring external perspectives as well. Lastly, the Logic Model does not include challenges in the PriCARE programme regarding PE, but they have been documented elsewhere by Danish et al.^29^ and Beland et al.^31^

## 5 CONCLUSION

The Patient Engagement in Health Implementation Research Logic Model will help academic researchers, healthcare providers, decision makers and patient partners involved or interested in PE in implementation research to plan and operationalize the resources and activities to achieve desired outcomes.

## AUTHOR CONTRIBUTIONS

Catherine Hudon, Maud‐Christine Chouinard, Kris Aubrey‐Bassler, Shelley Doucet and Vivian R. Ramsden contributed to the PriCARE research programme conception and design. Mathieu Bisson and Catherine Hudon led the different steps of the study. Mathieu Bisson drafted the interview guide with Jude Porter, Donna Rubenstein and Mike Warren. Mathieu Bisson analyzed the data. Mathieu Bisson, Catherine Hudon, Maud‐Christine Chouinard, Mireille Lambert and André Gaudreau interpreted the data with Kris Aubrey‐Bassler, Shelley Doucet, Vivian R. Ramsden, Olivier Dumont‐Samson, Dana Howse, Charlotte Schwarz, Alison Luke, Norma Rabbitskin, Jude Porter, Donna Rubenstein, Jennifer Taylor and Mike Warren. The first draft of the manuscript was written by Mathieu Bisson, Olivier Dumont‐Samson and Catherine Hudon, and all authors commented on subsequent versions of the manuscript. All authors read and approved the final manuscript.

## CONFLICT OF INTEREST STATEMENT

The authors declare no conflict of interest.

## ETHICS STATEMENT

This study was approved by Ethics Review Boards in each of the four participating provinces in this study: Comité d'éthique du Centre intégré universitaire de santé et services sociaux (CIUSSS) de l'Estrie‐CHUS; Research Ethics Boards Horizon Health Network; University of New Brunswick Research Ethics Board; Newfoundland and Labrador Health Research Ethics Board and Nova Scotia Health Research Ethics Board. All participants provided oral informed consent to participate in the study.

## Data Availability

The data that support the findings of this study are available on request from the corresponding author. The data are not publicly available due to privacy and ethical restrictions.
